# Hepatitis E Virus and rheumatic diseases: what do rheumatologists need to know?

**DOI:** 10.1186/s41927-020-00149-0

**Published:** 2020-09-21

**Authors:** Salvatore Di Bartolomeo, Francesco Carubbi, Paola Cipriani

**Affiliations:** 1 Rheumatology Unit, Department of Biotechnological and Applied Clinical Science, School of Medicine, University of L’Aquila, L’Aquila, Italy; 2 Department of Medicine, ASL1 Avezzano-Sulmona-L’Aquila, L’Aquila and Sulmona, Italy

**Keywords:** Hepatitis E virus, Rheumatic diseases, Immunosuppressive therapy, Chronic hepatitis, Rheumatic manifestations

## Abstract

**Background:**

Hepatitis E virus (HEV) represents the most common cause of acute hepatitis and jaundice in the world. About 2 million of infection cases occur each year in Europe, mainly as autochthonous anthropozoonosis, and HEV can be transmitted through undercooked pork meat. This infection has been linked to various extra-hepatic manifestations, while chronic infections with a rapid development of liver failure have been described in heavily immunosuppressed patients undergoing solid organ transplantations (SOTs), in patients with hematological diseases or with immunodeficiency virus infection.

**Main body of abstract:**

The purpose of this review article is to describe rheumatic manifestations related to HEV infection and their implications for rheumatologists in the daily clinical practice. Despite recent accumulating literature in this field, little is known about the course of the infection in patients with rheumatic diseases (RDs) and about the impact of immunosuppressive drugs. Moreover, HEV infection can mimic RDs’ manifestations or drugs toxicity. Specific guidelines on management are lacking and the majority of data are referred to SOTs receivers.

**Conclusions:**

More studies are needed to better understand the real impact of HEV infection in patients with RDs, regarding both clinical outcomes and their management.

## Background

Hepatitis E virus (HEV) is a non-enveloped, icosahedral, single-stranded positive RNA virus of 27–34 nm in diameter, that belongs to the *Hepeviridae* family [1, 2]. It was isolated for the first time in 1983 after an outbreak of fecal-oral transmitted non-A, non-B hepatitis among Soviet troops in Afghanistan [3], and in 1990 the virus genome was cloned [4]. It replicates in the cytoplasm of infected cells. Although HEV is a primary hepatotropic virus, infections of other tissues, including small intestine, colon, lymph nodes, neurons, kidney and placental tissue have been reported, partially explaining the pathogenesis of some extra-hepatic manifestations [5–7].

HEV has a 7.2-kb genome which is organized in three open reading frames (ORFs). ORF1 encodes non-structural proteins involved in the replication of viral genome (the so called “replicase”), including a methyl transferase, a putative protease, an RNA helicase and an RNA-dependent RNA polymerase. ORF2 encodes the viral capsid protein and ORF3 encodes a protein involved in release of viral particles from infected cells. Antibodies against the viral capsid are neutralizing [1, 7]. Virus in the stool, after biliary excretion, is non enveloped, while in the bloodstream it exists as “quasi-enveloped”, because virions are wrapped in membranes derived from infected cells after viral budding [7].

HEV human infections are caused by viruses belonging to the *orthohepevirus* genus that encompasses four species (A-D), of which mainly strains belonging to the specie A are pathogenic for humans; rat HEV (group C) has recently been described to cause acute and chronic infections in humans, although its real epidemiological impact is still unknown [8, 9]. A specie is, in turn, divided into 8 genotypes (GT1–8), but only GT 1–4 infect humans. The only exception was the isolation of the GT-7 in a man who consumed Camel milk [1, 6–10].

In particular, GT1 and GT2 are humans obligate pathogens spread by the fecal-oral route in less developed countries. GT3 and GT4 are the most prevalent strains in developed countries in which the infection is now considered mainly an anthropozoonosis, since pigs, wild boars and deers are considered the major reservoirs of the virus. Fruits, vegetables and seafood watered with water contaminated by infected manure could theoretically transmit the infection, but the real epidemiological impact of this route of infection is not known. Inter-human infection is rare, but it seems possible through fresh blood products from infected persons [1, 6–12].

HEV, in particular GT3, is now considered the most common cause of acute hepatitis in high-income countries where infection is acquired as a zoonosis with pigs and wild boars as primary hosts that, however, display no symptoms. In Europe, about 2 million new infections per year have been estimated to occur. The real HEV seroprevalence is probably underestimated, and in Europe there are some “hot spots” of high prevalence such as south of France, Pyrenees, Germany, Poland, Holland, Scotland. In Italy, a recent research of Lucarelli et al., found an high seroprevalence among blood donors in the Abruzzo region (central Italy). The prevalence was about 49%, likely related to the consumption of raw sausages since HEV shows the ability to survive in the dried and salty meat [7, 13, 14].

The clinical features of the infection are variables. The majority of cases are probably completely asymptomatic, identified only by anti-HEV seroconversion. In 5–10% of people a mild and self-limiting form resembling acute hepatitis A with fatigue, nausea, itching, jaundice and raising of liver function tests (LFTs) may occur, more frequently in males > 60 years of age. The Incubation period is about 3–8 weeks. Fulminant hepatitis whit acute liver failure (ALF) is rare, occurring in particular in patients with chronic liver diseases, and in about 0.5–3% of young adults. The infection during the third trimester of pregnancy can have up to 20–30% of mortality, but probably only for GT1 and GT2 genotypes [6, 7, 11].

Chronic infection is possible with GT3 and GT4 and it is defined as the persistence of detectable HEV-RNA in serum for more than 3 months, although others consider the limit of 6 months, in particular in immunocompetent patients [7, 11]. The risk of chronicity seems particularly high (> 50%) among solid organ transplantations (SOTs) receivers (SOTRs), due to anti-rejection treatments [15, 16]. Increasing evidence reveals risk among patients with immunodeficiency virus (HIV) infection and hematological conditions, in particular hematopoietic stem cells transplantation (HSCT), as well as rheumatic diseases (RDs) [7, 11, 17–20]. In these patients, chronic HEV infection can rapidly progress over 3–4 years to liver cirrhosis and ultimately liver failure [7].

Several data reveal that HEV infection (in particular GT3) is associated with several extra-hepatic manifestations, in particular neurological, but also renal, haematological, autoimmune and rheumatic conditions [7, 11, 21–24].

The purpose of this review article is to describe rheumatic manifestations related to HEV infection and their implications for rheumatologists in daily clinical practice. In particular, we designed a comprehensive literature search on this topic, by a review of reported published articles in indexed international journals until 30 November 2019, following proposed guidelines for preparing a biomedical narrative review [25].

## HEV as a cause of rheumatic manifestations

The precise pathogenesis of HEV-associated rheumatic manifestations (RMs) is largely unknown and probably multifactorial. HEV, like other pathogens, could act through molecular mimicy inducing autoimmunity in genetically predisposed hosts. A direct action of HEV on the immune system, as in other forms of reactive arthritis, is also possible, since HEV can replicate in various cell types. For example, in some cases of Guillan-Barrè Syndrome (GBS), HEV-RNA has been isolated from serum and cerebrospinal fluid with a “quasi-species” compartmentalization of neurotropic strains [7, 24, 26, 27]. Similarly to HCV, HEV can trigger the production of cryoglobulines; in particular, anti-HEV IgM and IgG and HEV-RNA was isolated in the cryoprecipitate of patients with cryoglobulinaemia [28]. HEV can, thus, trigger an autoimmune response that may explain the occurrence of mixed cryoglobulinaemia (MC) after viral clearance [28, 29].

Many infectious agents, including HEV, are believed to act as triggers in the pathogenesis of both cutaneous necrotizing small-vessel vasculitis (CNSVV) and Henoch-Schönlein Purpura (HSP). Vessel inflammation may occur as a consequence of direct viral replication, type II or immune complex-mediated (type III) reaction and/or cell-mediated retarded hypersensitivity response (type IV) [30]. In this context, viral eradication can be useful in reducing clinical manifestations. A reduction of proteinuria and an improvement of renal function after viral clearance (obtained either spontaneously or after medical treatment) has been reported in patients with glomerulonephritis associated with active HEV infection [7, 31].

HEV infection can be associated with various rheumatic complaints. Musculoskeletal involvement, such as arthralgia and myalgia can be present during acute hepatitis, even in the absence of jaundice [7, 32–35]. Association with acute polyarthritis [36, 37], that probably represent a form of reactive and self-limiting arthritis, has also been described.

Although cryoglobulinaemia is associated with hepatitis C virus (HCV), many recent reports describe the association between HEV and MC (Type II or III). MC seems to occur in patients with chronic infection, in particular SOTRs under immunosuppressive treatments, although Guinautl at al. described a case of MC in a non-immunocompromised patient with HEV infection. Clinical manifestations range from mild to severe forms [7, 21, 24, 36–38], with or without renal and musculoskeletal involvement. Moreover, a case of HEV-associated cryoglobulinaemia with myalgias, rash, arthralgia has been reported also in a liver transplant recipient [39].

One case of HSP occurred in a 6 years old girl with an acute HEV infection [40], while a case of CNSVV has been described in an adult with evidence of HEV infection [30]. Both cases had a favorable and self-limiting course requiring only supportive therapy with the resolution of vasculitis after the viral clearance. HEV infection has also been associated with myocarditis [41], autoimmune thyroiditis [42], autoimmune hepatitis (where HEV could probably either trigger the development of autoimmunity or simulate both clinical and serological features of autoimmune hepatitis) [43–46], multiplex mononeuropathy, Parsonage-Turner Syndrome, GBS and other neurological manifestations [7, 24].

Specific guidelines for the management of HEV-related RMs are lacking and therapeutic choices depend on clinical severity. As their course is frequently self-limiting, patients might only need symptomatic therapies. Eradication of the virus, through either reduction of the immunosuppressive therapy, where feasible, or administration of antiviral treatments (e.g. ribavirin), could speed up the recovery of patients, as demonstrated in HEV-associated glomerulonephritis. However, in refractory and most severe cases, specific immunosuppressive treatment as for non HEV-induced RMs is needed, in adjunction to viral eradication, according to the relevant guidelines for each disease [7, 24, 28, 31].

## HEV in patients with a rheumatic disease

HEV infection can occur in patients with RDs leading to different possible consequences.

The infection can simulate a flare of the disease leading to an unnecessary, and potentially harmful, increase of the immunosuppressive therapy [7, 32–37].

Moreover, the alterations in LFTs due to the infection should be differentiated from those of other etiology that can occur in patients with rheumatic disease such as drug toxicity (e.g. Methotrexate (MTX)), liver involvement by the rheumatic disease itself, concomitant autoimmune hepatitis, infections (of either opportunistic or non-opportunistic etiology) [10, 47–51]. In particular, recently we reported a case of a woman, affected by rheumatoid arthritis (RA) who developed fatigue, diffuse itching and increased LFTs soon after the introduction on leflunomide (LEF) and that, subsequently, resulted positive for acute HEV infection. LEF was stopped and restarted after 5 months, when viral clearance has occurred. LFTs remained normal ruling out the possibility of drug toxicity [34].

The high risk of developing chronic infections with a rapid progression to liver cirrhosis among SOTRs and other heavily immunosuppressed patients is well know and represents a clinical challenge [7, 15, 16, 52]. In the last years, patients with rheumatic diseases are increasingly treated with biological disease-modifying antirheumatic drugs (b-DMARDs) or targeted synthetic (ts-) DMARDs with or without conventional synthetic (cs-) DMARDs. The risk of HBV and HCV reactivation during immunosuppressive treatment with cs-, b- or ts-DMARDS and corticosteroids for various RDs is well established [53, 54].

Despite the increasing evidence on HEV infection in rheumatic diseases, the course of the disease has not been fully elucidated.

We found a total of 60 cases of HEV infection (Table 1) occurring in immunosuppressed patients, treated with DMARDs, other than SOT receivers,HIV carriers or having undergone HSCT [20, 28, 55–59].

**Table 1:** Cases of HEV infection in patients with autoimmune diseases and treated with DMARDs

No.pt^REF^	Gender/age	RD	b/tsDMARD/withdrawal	csDMARD/ withdrawal	PDN (mg/d)	HEV Gt	Ribavirin	HEV-RNA clearance (weeks)	Evolution of HEV infection
1 [47]	M/60	RA	IFX/NA	MTX,BUC/NA	yes (dose NA)	4	NO	NR	died due to FE
2 [47, 49]	F/69	RA	ABA/ YES	LEF/ Yes	5	3	NO	NR	Improved
3 [47, 49]	M/55	RA	RTX/ YES	MTX/ Yes	YES (NA)	3	YES	12	improved
4 [47, 49]	F/62	RA	IFX/ YES	MTX/ Yes	NO	NA	NO	4	improved
5 [47, 49]	M/72	RA	RTX/ NO	MTX,LEF/ Yes	NO	NA	NO	NR	improved
6 [47, 49]	F/49	RA	TCZ/ YES	MTX/ Yes	3	3f	NO	6	ALF,improved
7 [47, 49]	F/69	RA	ABA/ YES	LEF/ Yes	5	3f	NO	7	improved
8 [47, 49]	M/69	RA	RTX/ NO	MTX/ No	NO	NA	YES	10,5	improved
9 [47, 49]	M/61	RA	RTX/ NO	LEF/ Yes	3	NA	NO	8	ALF,improved
10 [47, 49]	F/53	RA	ABA/YES	MTX/ Yes	NO	NA	NO	9	improved
11 [47, 49]	F/44	RA	RTX/ YES	MTX/ Yes	NO	3c	NO	9,5	improved
12 [47, 49]	F/55	RA	ETN/ YES	MTX/ Yes	NO	NA	NO	4	improved
13 [47, 49]	F/60	RA	ADA/ YES	MTX/ Yes	4	3f	NO	8	improved
14 [47, 49]	M/59	RA	TCZ/YES	MTX/ Yes	7	NA	NO	4	improved
15 [47]	F/68	RA	n/a	MTX/ Yes	5/weekly	NA	NO	5,7	improved
16 [47]	F/33	RA	TCZ/ YES	n/a	NO	NA	NO	NR	improved
17 [47]	F/64	RA		MTX, BUC/NA	NO	3	NO	NR	improved
18 [47]	F/74	RA	TOF/ YES	n/a	YES (NA)	3	NO	NR	improved
19 [47]	F/52	RA		MTX/NA	NO	3	NO	NR	improved
20 [47]	F/51	RA	RTX/ YES	n/a	NO	NA	YES	8	improved
21 [15, 47]	F/58	RA		ACT,BUC,MIZ/ No	5	NA	NO	NR	improved
22 [15, 47]	M/61	RA	ETN/ YES	MTX/ Yes	3	NA	NO	NA	improved
23 [15, 47]	M/67	RA		MTX,TAC/ Yes	5	NA	NO	NR	improved
24 [15, 47]	F/52	RA		MTX,MIZ,TAC/ Yes	4	NA	NO	NR	improved
25 [47]	M/63	RA	ADA/ YES	MTX/ Yes	3	3	YES	6 wks after ribavirin	chronic infection then improved
26 [47]	F/63	RA	TCZ/ YES	n/a	3	3e	NO	6	Improved
27 [23]	F/39	RA		LEF/ Yes	10	3	NO	About 7,8	Improved
28 [48]	F/65	pSS	RTX/ YES	MMF/ Yes	NA	3	YES	NR	Chronic infection
29 [49]	F/30	JIA	IFX/ YES	MTX/ Yes	6	3f	YES	4	Impreved
30 [49]	F/54	AS	ADA/ NO	n/a	NO	NA	NO	4	Improved
31 [49]	M/62	PsA		MTX/ Yes	NO	3c	YES	7	Improved
32 [49]	F/52	AS	INF/ NO	MTX/ No	NO	NA	YES	3	Improved
33 [49]	F/25	PsA		MTX/ Yes	20	NA	NO	NR	Improved
34 [49]	M/70	UA		MTX/ Yes	5	3c	NO	6	Improved
35 [49]	F/30	PsA		CyA/ Yes	NO	3f	YES	7,5	Improved ^c^
36 [49]	M/38	AS	IFX/ YES	LEF/ No	4,5	NA	NO	NR	improved
37 [49]	M/40	AS	ETN/YES	n/a	NO	NA	NO	NR	improved
38 [49]	F/79	JA		MTX/ Yes	2	NA	NO	NR	improved
39 [49]	M/35	AS	INF/ YES	MTX/ Yes	NO	NA	NO	NR	improved
40 [49]	M/61	PsA	ADA/ YES	n/a	NO	NA	NO	NR	improved
41 [50]	M/57	RA	RTX/ NO	MTX/ No	NO	NA	NO	11	improved
42 [50]	F/69	RA	TNFi^a^/ NO	MTX/ No	NO	NA	NO	4	improved
43 [50]	F/56	SLE	TNFI^a^/ NO	MTX/ No	NO	NA	NO	4	improved
44 [50]	F/65	RA		MTX/ Yes	NO	1	NO	4	improved
45 [50]	F/75	RA		MTX/ No	NO	NA	YES	18	improved
46 [50]	M/67	PsA	TNFI^a^/ NO	n/a	NO	NA	YES	11	improved
47 [50]	M/58	ECD	TNFI^a^/ YES^b^	MTX/ Yes^b^	NO	NA	NO	3	improved
48 [50]	M/59	GRA		CYC/ No	NO	NA	NO	4	improved
49 [50]	F/51	RA	ABA/ YES	n/a	NO	NA	NO	16	Improved
50 [50]	F/30	JIA	TNFi^a^/ YES	MTX/ Yes	NO	NA	YES	4	Improved
51 [50]	F/54	PsA	TNFi^a^/ NO	n/a	NO	NA	NO	5	Improved
52 [50]	M/62	PsA		MTX/ Yes	NO	NA	YES	7	Improved
53 [50]	F/52	AS	IFX/ YES	n/a	NO	NA	NO	3	Improved
54 [50]	F/25	PsA	TNFi^a^/ YES	MTX/ Yes	NO	NA	NO	< 1	Improved
55 [50]	M/70	UA		MTX/ Yes	YES (NA)	NA	NO	6	Improved
56 [50]	M/29	GRA		MMF/n/a	7,5	NA	YES	48	clearance riba†
57 [50]	M/34	RF		SIRO/ No	60	NA	YES	96	riba clearance†
58 [50]	M/55	PsA	TNFi^a^/ YES	n/a	NO	NA	NO	3	Improved
59 [52]	M/33	CD/PSC	ADA/ NO	6-MP/ Yes	5	1	YES	about 24	clearance riba†
60 [51]	F/60	UC	INF/ NO	MES/ Yes	10	3a	NO	about 2	Improved

List of abbreviations: *ABA* Abatacept, *ACT* Actarit, *ADA* Adalimumab, *ALF* Acute liver failure, *AS* Ankylosing spodilytis, *BUC* Bucillamine, *CD* Crohn’s disease, *DMARDs* Disease modifiyng antirheumatic drugs, *bDMARDs* Biologic DMARDs, *csDMARDs* Conventional synthetic DMARDs, *ECD* Erdheim Chester disease, *ETN* Etanercept, *FE* Fulminant hepatitis, *FO* Follow-up, *GRA* Granulomatosis, *HEV Gt* Hepatitis e genotype, *IFX* Infliximab, *JA* Jaccoud arthtropathy, *JIA* Juvenile idiopathic arthritis, *LEF* Leflunomide, *MIZ* Mizoribine, *MTX* Methotrexate, *NA* Not available, *n/a* Not applicable, *NR* Not reported, *PRD* Prednisone, prednisolone, *Psa* Psoriatic arthtritis, *PSC* Primary sclerosing cholangitis, *pSS* Primary Sjogren’s syndome, *RA* Rheumatoid arthtritis, *RF* Retroperitoneal fibrosis, *RTX* Rituximab, *SIRO* Sirolimus, *SLA* Systemic lupus eritematosus, *TAC* Tacrolimus, *TCZ* Tocilizumab, *TOF* Tofacitinib, *UA* Undetermined arthtritis, *UC* Ulecrative colitis, *wk.* Week. ^a^*TNFI* Tumor necrosis factor inhibitor not specified; ^b^ not specified the degree of reduced immunosuppression; ^c^developed bilateral Parsonage-Turner syndrome

^†^HEV-RNA clearance after introduction of ribavirin

In a recent literature review, 26 cases of HEV infection occurred during the treatment of RA [55]. Twenty patients were treated with ts- or b-DMARDs with or without cs-DMARDs, 6 patients with cs-DMARDs alone, namely MTX, LEF, bucillamine, mizoribine, actarit and tacrolimus (TAC) while low-dose steroids were used in 16 cases. Interestingly in the majority of cases HEV clearance and LFTs improvement occurred soon after the withdrawal of DMARDs without adding anti-viral therapy. One patient died because of acute liver failure and one patient, who continued adalimumab therapy, developed a chronic infection that resolved after adalimumab withdrawal and ribavirin treatment. In other 3 cases ribavirin was used with a rapid eradication of the virus (< 3 months), interestingly these patients were receiving Rituximab (RTX) with or without MTX [55]. In a French multicenter retrospective study among patients with various chronic inflammatory arthritis, 23 cases of HEV infections were reported [56]. Treatments included discontinuation of immunosuppressant in 20 patients and ribavirin treatment in 5 patients (1 with RA, 1 with Juvenile idiopathic arthritis, 2 with Psoriatic arthritis (PsA), 1 with ankylosing spondylitis). None of the patients developed chronic infection and/or fulminant hepatitis, 1 patient with PsA developed bilateral Parsonage-Turner Syndrome (#35) [56].

A recent European multicenter retrospective Cohort study reported 21 cases of HEV infections among patients with different systemic diseases, including RA (*n* = 5), PsA (*n* = 4), other variants of chronic arthritis (n = 4), primary immunodeficiency (*n* = 3), systemic granulomatosis (*n* = 2), systemic lupus erythematosus (*n* = 1), Erdheim–Chester disease (n = 1), and retroperitoneal fibrosis (n = 1) [57]. Chronic infection (lasting> 3 months) developed in seven (33%) patients. In particular, they reported the development of chronic infection in two (40%) patients with RA, 3 (100%) with primary immunodeficiency, one (100%) with retroperitoneal fibrosis and one patient (100%) with systemic granulomatosis. They were treated with MTX monotherapy (*n* = 2), Mycophenolate Mofetil (MMF)/prednisone (*n* = 1), sirolimus/prednisone (n = 1). Overall 38% cleared the infection after reduction of the immunosuppressant therapy, 52% after treatment with ribavirin (no standard dose and/or regimen were used), one patient was lost at follow-up (5%), and one patient (5%), with primary immunedeficiency, relapsed after initial successful response to ribavirin (#49). No patients died from hepatic complications [20].

Abignano et al. described a patient treated with RTX and MMF for Sjögren’s syndrome who developed a chronic HEV infection. Anti-HEV IgM and IgG were both negative, probably due to the effect of RTX on the humoral immunity, but HEV-RNA was positive in the stool. HEV-RNA disappeared after introduction of ribavirin, but the infection relapsed after ribavirin suspension [57].

Our knowledge is limited about HEV infection in the field of inflammatory bowel diseases. Recently two case reports have been published. One case of a self-limiting infection in a patient with ulcerative colitis [58], and 1 unusual case of HEV GT1 infection in a patient with Chron’s disease treated with mercaptopurine, adalimumab and prednisolone who seems to have developed a prolonged course (however in this case, chronic infection is not well documented) [59, 60].

No data are currently available about a possible role of HEV in causing Osteoporosis and/or Osteomalacia.

## Treatment of HEV infection in patients with RDs

In the case of HEV infection occurring during immunosuppressive treatment for RDs, specific guidelines developed for rheumatic patients, are lacking. However, data are accumulating and the outcome seems favorable in the majority of cases [20], but chronicity can occur [57]. In mild and self-limiting infections (with HEV-RNA clearance within 3 months), only supportive measures are needed with a thorough follow-up. It could be useful, if feasible, to reduce the immunosuppressive therapy for a short while in order to promote viral clearance [34]. In cases of severe acute hepatitis, treatment with ribavirin could rapidly improve liver function (these data come from studies in immunosuppressed patients with other diseases) [7]. Corticosteroids have been added in cases of ALF with positive effects, however more data are needed [7, 61].

In the case of chronic infection (with HEV-RNA being detectable for over 3 months), it could be advisable to follow the guidelines for SOTRs or for hematological patients proposed by Mallet et al. [7, 62] and discontinue and/or reduce the DMARDs, if feasible. This approach led to viral clearance in about 1/3 of cases of HEV in SOTRs [1, 7].

Subsequently, according to the patient characteristics, the introduction of ribavirin could be considered. Several different schemes of treatment have been used in the past [59], but the one recently proposed by the European Association for the Study of the Liver (EASL) practice guidelines (600 mg/d for 12 weeks) seems preferable since it allowed to achieve sustained virological response (SVR) in 78% of SOTRs. In cases of early relapse, a 6-month course of ribavirin can be used. In ribavirin resistant and/or intolerant patients, treatment is difficult. A 3-month course of PEGylated-interferon-a has been used in liver transplant patients. However, it could be harmful in autoimmune diseases and so its usefulness is dubious. Sofosbuvir has some anti-HEV actions in vitro, however, in vivo results are conflicting [7, 63].

## Practical considerations

HEV is a ubiquitous virus with two distinct epidemiologic features: i. GT1 and GT2 is characterized by outbreaks in endemic areas (e.g. Asia), mainly causes acute infections and is linked to the consumption of inadequately sanitized water; ii. GT3 and GT4 is more spread in developed countries were it represents an autochthonous anthropozoonosis [1, 6, 7, 34].

,HEV infection incidence in Europe has an average rate of 19.16% (7.5–31.9%) [14], probably underestimated, due to high frequency of asymptomatic cases and the lack of its awareness by physicians. A recent review article reported a seroprevalence ranging from 0.12 to 49% in Italy, probably due to variability in test sensitivity and dietary habits [64]. In particular, in the Abruzzo region the high prevalence of the infection, caused by GT3, seems to occur as a locally acquired anthropozoonosis related to the ingestion of locally-bred undercooked pork meat [13, 64, 65].

**Fig. 1:**
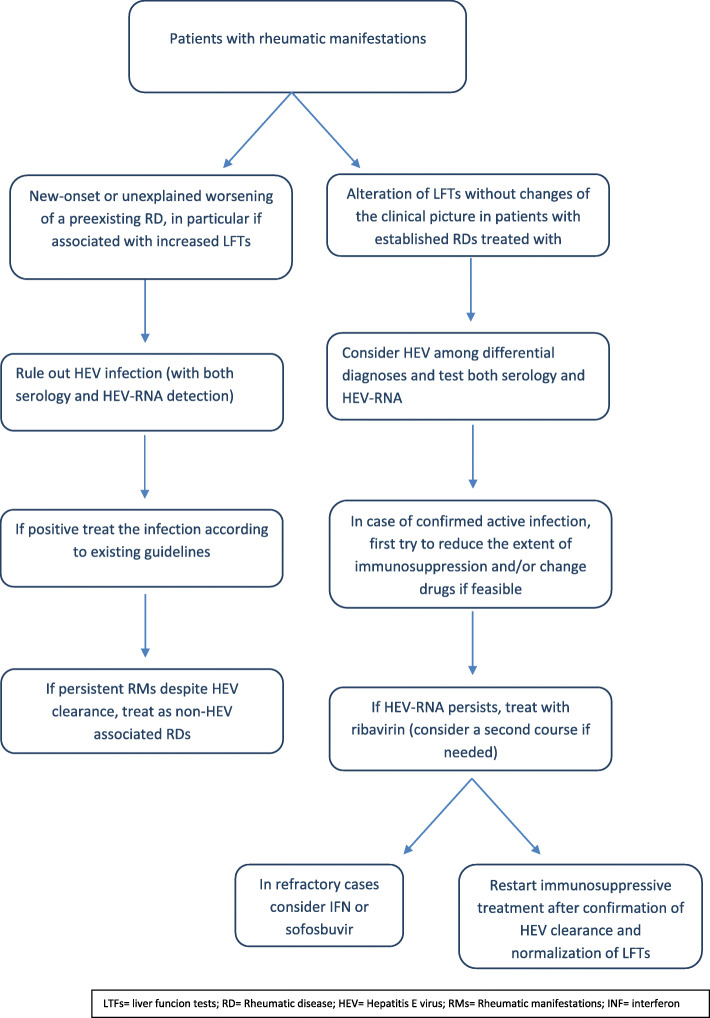
Diagnostic and therapeutic algorithm in patients with rheumatic manifestations. List of abbreviations**:** LTFs = liver funcion tests; RD = Rheumatic disease; HEV = Hepatitis E virus; RMs = Rheumatic manifestations; INF = interferon

HEV can have a profound impact in the rheumatologic daily practice, and we do not completely know the course of the infection in patients with RDs and/or under immunosuppressive therapy [34]. However, we suggest some practical indications (Fig. 1):

1. Considering and searching for HEV during the initial diagnostic workup of a patient with a suspect of reactive arthritis or a possible extra-hepatic manifestation of HEV, in particular if accompanied by abnormality in LFTs.
2. Thinking about HEV in the differential diagnosis of a patient receiving drugs for a rheumatic disease, with persistent elevation in LFTs or with acute hepatitis. This could allow to rule out drug-induced liver injury thus avoid the withdrawal of an effective treatment, as it was the case for our patient and in others case reports [12, 34, 49], or prevent an unnecessary strengthening of the immunosuppressive therapy for an erroneous diagnosis of autoimmune hepatitis.
3. When HEV infection occurs during immunosuppressive therapy, it is important to test such patients for both antibodies and HEV-RNA in the blood and in the stool because immunocompromised patients could not be able to produce detectable antibodies or can have a delayed seroconversion, leading to false negative results when only serological methods are used [1, 7, 52]. Moreover, the outcome of patients varies according to the type of immunosuppressants used, with the more powerful regimens carrying the highest risk of chronicity. In particular, calcineurin and mTOR inhibitors seem to stimulate, while mycophenolic acid could inhibit viral replication and corticosteroids should not interfere with viral clearance [1, 7]. In highly selected cases, it could be reasonable, where possible, to change DMARD, e.g. switching from a calcineurin or mTOR inhibitor to MMF due to its anti-viral in vitro effect, however in vivo data are lacking.

There are not specific predictive factors for chronicity in rheumatic patients. Prior studies in SOTRs have found an increased risk with a more profound immunosuppression characterized by reduced levels of CD2, CD3, CD4, use of TAC, lower serum levels of interleukin-1 receptor antagonist and interleukin-2 receptor, and increased serum concentration of chemokines involved in liver leukocyte recruitment, such as RANTES, MIP-1, MCP-1 and CXCL8 [1]. GT4 seems to be more virulent than Gt3 [55].

Teeatment with TAC and presence of thrombocytopenia were independent predictive factors for chronic HEV infection in SOTRs in a previous multicentre study [1, 66]. In addition, transplanted patients who developed chronic disease have had greater heterogeneity of HEV as a “quasi-species”, in relation to those that had spontaneous resolution [1, 67]. These observations are not specifically referred to rheumatic patients and the risk likely varies according with the diverse RDs that carry different immunological impairment.

Close monitoring of the patient is advisable, in order to promptly introduce the anti-viral therapy and to monitor treatments’ outcome.

An infectious diseases specialist should be consulted for the tailoring of treatment and a shared management of the patient. Finally, DMARDs can be safely reintroduced after confirmation of virus clearance and recovery of the liver function [34].

Prevention of HEV infection is complex*.* In vitro data suggest that HEV can be inactivated by heating at 70 °C for more than 2 min or at 80 °C for one minute, while at room temperature it can remain active for up to 28 days [7, 68], so it could be useful to avoid undercooked pork meat in high risk patients from highly endemic regions. A vaccine against HEV was licensed in China in 2011 showing an efficacy of 97% in preventing acute hepatitis. It is a recombinant vaccine produced by a portion of the ORF 2 of the Gt1 and induces neutralizing antibodies that persist up to 4–5 years. It is administered with a 3 dose schedule (0, 1, and 6 months). Its capability to prevent also Gt4 symptomatic infection may suggest a cross-reactivity. However, it is not licensed in Europe and USA ad clinical trials are ongoing [1, 7, 68–70].

In Italy, blood donors screening for HEV is not routinely performed in the daily clinical practice (like in other countries) and an extensive screening might be very expensive. However, such approach could be advisable, at least in high endemic regions, for blood products to be used in immunocompromised patients at higher risk of infection.

## Conclusion

HEV infection is increasingly recognized among rheumatologic patients. Clinicians should think about it in the presence of liver damage or in the context of a possible extrahepatic manifestation occurring with LFTs abnormalities, in particular in high prevalence areas. Further studies are needed to better understand the course of the infection in such patients and to develop specific management guidelines. Moreover, future studies should evaluate the utility of screening programs in patients needing to be treated with DMARDs and assess the cost effectiveness of preventive measures such as avoiding undercooked pork meat, vaccination, and extensive screening of blood products.

## Data Availability

Not applicable.

## Abbreviations

ALF: Acute liver failure
AS: Ankylosing spondylitis
b: Biologic
CNSVV: Cutaneous necrotizing small-vessel vasculitis
cs: Conventional synthetic
DMARDs: Disease modifying antirheumatic drugs
EASL: European Association for the Study of the Liver
GBS: Guillian-barrè syndrome
Gt: Genotype
HCV: Hepatitis C virus
HEV: Hepatitis E virus
HIV: Human immunodeficiency virus
HSP: Henoch-Schönlein Purpura
INF: Interferon
LEF: Leflunomide
LFTs: Liver function tests
MC: Mixed cryoglobulinaemia
MMF: Mycophenolate mofetil
MTX: Methotrexate
ORF: Open reading frames
PsA: Psoriatic arthtritis
RA: Rheumatoid arthritis
RDs: Rheumatic diseases
RMs: Rheumatic manifestations
RTX: Rituximab
SOTs: Solid organ transplants
SOTRs: Solid organ transplantation receivers
SVR: Sustained virological response
TAC: Tacrolimus
ts: Targeted synthetic
